# Considerations for quality assurance of multiplex malaria antigen detection assays with large sample sets

**DOI:** 10.1038/s41598-021-92723-w

**Published:** 2021-06-24

**Authors:** Rachel Alvarado, Lotus L. van den Hoogen, Nnaemeka C. Iriemenam, Oluwaseun O. Akinmulero, Andrew N. Thomas, Israel Tamunonengiyeofori, Evbuomwan Erasogie, Achugbu C. Chimaoge, Ayuba B. Dawurung, Mudiaga K. Esiekpe, Mary U. Okoli, Nwando Mba, Abiodun Ogunniyi, Alash’le Abimiku, Mark Maire, Orji O. Bassey, McPaul Okoye, Mahesh Swaminathan, Stacie M. Greby, Nnaemeka Ndodo, Chikwe Ihekweazu, Ado Abubakar, Laura Steinhardt, Eric Rogier

**Affiliations:** 1 Malaria Branch, Division of Parasitic Diseases and Malaria, Centers for Disease Control and Prevention, Atlanta, GA USA; 2 Center for Applied Malaria Research and Evaluation, Tropical Medicine Department, Tulane University School of Public Health and Tropical Medicine, New Orleans, LA USA; 3 Division of Global HIV and TB, Centers for Disease Control and Prevention, Abuja, Nigeria; 4 Institute of Human Virology (IHVN), Central Business District, Abuja, Nigeria; 5 Nigeria Centre for Disease Control (NCDC), Abuja, Nigeria

**Keywords:** Microbiology techniques, Immunological techniques, Parasitology, Policy and public health in microbiology

## Abstract

Multiplex assays for malaria antigen detection can gather data from large sample sets, but considerations for the consistency and quality assurance (QA) of mass testing lack evaluation. We present a QA framework for a study occurring November 2019 to March 2020 involving 504 assay plates detecting four *Plasmodium* antigens: pan-*Plasmodium* aldolase and lactate dehydrogenase (LDH), histidine-rich protein 2 (HRP2), *P. vivax* LDH (PvLDH). Controls on each plate included buffer blank, antigen negative blood, and 4-point positive dilution curve. The blank and negative blood provided consistently low signal for all targets except for pAldolase, which showed variability. Positive curve signals decreased throughout the 5-month study duration but retained a coefficient of variation (CV) of < 5%, with the exception of HRP2 in month 5 (CV of 11%). Regression fittings for inter-plate control signals provided mean and standard deviations (SDs), and of 504 assay plates, 6 (1.2%) violated the acceptable deviation limits and were repeated. For the 40,272 human blood samples assayed in this study, of 161,088 potential data points (each sample × 4 antigens), 160,641 (99.7%) successfully passed quality checks. The QA framework presented here can be utilized to ensure quality of laboratory antigen detection for large sample sets.

## Introduction

Detection of *Plasmodium* antigens in blood samples has proved to be effective for identifying a person’s infection status^1,2^. In clinical applications, malarial antigen detection can be used as an indication of disease severity, and is also used widely for fast diagnostic testing through malaria rapid diagnostic tests (RDTs)^2–4^. In the laboratory setting, tests for malaria antigens extend past a simple binary result and enable a sensitive measure to quantify antigen concentrations for research purposes^5–8^.

Different laboratory techniques can be used to assay for malarial antigens including enzyme-linked immunosorbent assays (ELISAs) and the multiplex bead-based immunoassay platform^9^. Malaria antigen detection by conventional sandwich ELISA has been in use for decades, but contemporary multiplex assays have shown promise by exhibiting lower limits of detection and requiring less sample volume for collection of data for multiple antigens^10,11^. Recently, our group designed a bead-based immunoassay using the Luminex xMAP^®^ platform for antigen detection of pan-*Plasmodium*, *P. falciparum,* and *P*. *vivax*-specific targets to a single panel^8,12,13^.

Quality assurance of immunoassays using controls and standards is imperative to assess variability and reliability across samples or batches. The World Health Organization’s (WHO) National Institute for Biological Standards and Control (NIBSC) has developed an international serological standard for *P. falciparum* IgG detection^14^, and quality assurance schemes for high-throughput antibody detection have been published^15^. Our group previously published a quality assurance scheme for large-scale serosurveys by analyzing various controls for IgG responses to identify aberrations and variation in standard curve parameter estimates within and between periods of sample collection^16^. No international standards currently exist for detection of multiple malaria antigens via laboratory immunoassays, though different assays have been validated for multiplex antigen detection^8,17^.

Here, we discuss the quality assurance elements used to ensure reliable malaria antigen data throughout a large-scale multiplex antigen detection study (N = 40,272 samples tested among 504 assay plates) performed between November 2019–March 2020. We outline a quality assurance scheme for inter- and intra-plate variability for malarial antigens and include sample level assessments to identify aberrant samples that require repeating to affirm the reliability of the data^8^.

## Materials and methods

### Test samples from Nigeria serosurveillance study

Samples for a multi-disease serosurveillance study were from the Nigeria HIV/AIDS Indicator and Impact Survey (NAIIS), a large nationally-representative household survey implemented from July–December 2018 in Nigeria [NAIIS Technical Report, see footnotes]. Written informed consent/parental informed consent and assent (for a child 10–17 years) was obtained from all participants included in NAIIS. Plasma and dried blood spot (DBS) samples were collected and stored in a biorepository at the Nigerian Centre for Disease Control National Reference Laboratory (NRL). Samples were included in this study only if participants provided informed consent/parental permission for storage and future testing of their biological samples, and only the DBS were assayed here. The study was determined to be public health surveillance and non-human subjects research by CDC, determined IRB review was not required by University of Maryland Baltimore, and provided a research exemption by the Nigerian National Health Research Ethics Committee (NHREC).

### Standard controls

Three types of controls were included on each assay plate: a no sample blank (consisting of only elution buffer), negative human blood at 1:20 dilution (the same concentration as test blood samples being assayed), and a 4-point antigen dilution curve. The negative human blood control was created by pooling a 1:20 blood dilution for 100 pre-screened Nigerian DBS samples that were found to be malaria antigen negative. The first point of the antigen dilution series was created by pooling recombinant antigens: pAldolase (Pv2 clone); PfLDH; PvLDH; HRP2 (Type B)^8,17^. The recombinant PfLDH, PvLDH, and HRP2 were all from MicroCoat (Starnberger See, Germany). The *P. vivax* Pv2 aldolase antigen was expressed and purified as described previously^8^. The second point of the positive curve was a four-fold dilution of the first point, and serial fourfold dilutions created the third (1:16) and fourth (1:64) points. For all three types of controls, large batches were prepared at the beginning of the study and stored at 4 °C with the intent of utilizing the same preparation throughout the entire course of data collection. Complete standard curves for pLDH, PvLDH, and HRP2 were prepared three times throughout the duration of data collection at months 1, 3, and 5 of data collection.

### Multiplex antigen detection assay

The bead-based multiplex assay for malaria antigen detection was performed as described previously^8,17^ at the NRL. Magnetic microbeads (xMAP, Luminex Corp.) were covalently bound to antigen capture antibodies by the Luminex antibody coupling kit according to manufacturer’s instructions. For one milliliter of microbeads (12.5 × 10^6^ beads) antibody coupling concentrations were: anti-pan-*Plasmodium* aldolase antibody (pAldolase, 12.5 μg, Abcam); anti-pan-*Plasmodium* lactate dehydrogenase antibody (pLDH, 12.5 μg of clone M1209063, Fitzgerald); anti-*P. vivax* LDH antibody (PvLDH, 12.5 μg of clone M1709Pv2); anti-*P. falciparum* histidine-rich protein 2 (HRP2, 20 μg, clone MPFG-55A, ICLlabs). Detection antibodies were also prepared in advance by biotinylating (EZ-link Micro Sulfo-NHS-Biotinylation Kit, linker of 13.5 angstroms, ThermoScientific) according to manufacturer’s instructions. Final prepared dilution of detection antibodies was 1.0 mg/mL and for anti-malarial antigen specific antibodies as follows: pAldolase (Abcam), pLDH and PvLDH (1:1 antibody mixture of clones M1709Pv1 and M86550, Fitzgerald), HRP2 (1:1 antibody mixture of MPFG-55A and MPFM-55A, ICLlabs). All reagents were stored at 4 °C until use in the immunoassay.

To rehydrate blood samples, a 6 mm filter paper punch from each DBS (approximately 10 μL whole blood) was placed into 400 μL of elution buffer: PBS pH 7.2, 0.3% Tween-20, 0.5% casein, 0.5% BSA, 0.5% polyvinyl alcohol, 0.8% polyvinlypyrrolidine, 0.02% sodium azide, and 3 μg/mL of *E. coli* lysate (to prevent nonspecific binding). This provided a 1:40 dilution of whole blood which was used for the immunoassay.

Assay reagents were diluted in buffer: containing 0.1 M phosphate buffered saline (PBS) pH 7.2, 0.05% Tween-20, 0.5% bovine serum albumin (BSA), and 0.02% sodium azide. For all wash steps, assay plate was affixed to a handheld magnet (LuminexCorp), and gently tapped for 2 min to allow bead magnetization before evacuation of liquid and washing with 150 μL PBS, 0.05% Tween-20. The four bead regions were combined in dilution buffer (in a reagent trough) and pipetted onto a 96-well assay plate (BioPlex Pro, BioRad) at a quantity of approximately 800 beads/region. Plates were washed twice, and 50 μL of controls or samples pipetted into appropriate wells. Following 90-min gentle shaking at room temperature protected from light, plates were washed 3 ×. A mixture of detection antibodies was prepared in dilution buffer (pAldolase at 1:2000, all others at 1:500), and 50 μL added to each well for a 45-min incubation. After 3 washes, 50 μL of streptavidin–phycoerythrin (at 1:200, Invitrogen) was added for a 30-min incubation. Plates washed 3 ×, and 50 -μL dilution buffer added to each well for 30-min incubation. Plates washed once and beads resuspended in 100 mL PBS. After brief shaking, plates were read on MAGPIX machine (Luminex Corp) with target of 50 beads per region. The median fluorescence intensity (MFI) value was generated for all beads collected for each region by assay well and subtracting the assay signal from wells with dilution buffer blank provides an MFI-background (MFI-bg) value used for analyses.

As the positive and negative controls were needed for direct comparison of consistency among assay plates in the study, if plates were missing data for the buffer blank or for more than one point in the positive dilution series, that assay plate was automatically selected for repeating.

### Statistical analysis

All statistical analyses were performed in R version 3.6.3 (R Foundation for Statistical Computing, Vienna, Austria). First, assay plates that had standard signal values falling outside an acceptable range of variation during assay processing were identified. For this, a self-starting exponential decay function (Formula 1; for HRP2 and pAldolase) or 3-parameter logistic regression function (Formula 2; for LDH and PvLDH) was fitted using MFI measurements for the first point of the 4-point antigen dilution curve (1:1 dilution) and plate number. Regression functions were applied to MFI measurements in the first point of the 4-point antigen dilution curve over time (i.e., plate number). Forty-one repeated plates due to missing control data were included at the end (Table 1).

1 $$ y(x) = yf + (y0 - yf)e^{{ - \alpha x}} $$

where *x* is plate number and the measured MFI in the first point of the 4-point antigen dilution curve (*y*) starts at *y*0 and decays towards *yf* at a rate *α*.

2 $$ y(x) = ~\frac{{Asymp}}{{1 + e^{{\frac{{(xmid - x)}}{{scal}}}} }} $$

where *x* is plate number and *y* is the measured MFI in the first point of the 4-point antigen dilution curve, *Asymp* is the top asymptote, *xmid* is the *x* value corresponding to the inflection point, and *scal* is the Hill slope. The model that resulted in the lowest Akaike information criterion (AIC) was used; if one of the models did not converge, the alternative was used. Next, residual plots were created and plates that fell outside of the ± 2 standard deviations (SD) range for pLDH and HRP2 were repeated. Secondly, inter-plate variability was assessed using MFI measurements from the complete 4-point antigen dilution curve. Only plates with MFI measurements available for all four dilution points of the curve were included (Table 1). For this, MFI measurements were log10-transformed, dilution step was coded 1 through 4, and 3-parameter logistic regression was fitted (Formula 2) where denotation is the same as described above except for *x* which is dilution step. To construct the fitted 4-point antigen dilution curves, the mean, 2.5th and 97.5th percentile for fitted values was extracted across all plates by month of data collection to adjust for the decay in MFI measurements seen in the positive control over time. In addition, the *xmid* coefficient was extracted for each plate and the coefficient of variation calculated (SD/mean multiplied by 100%) to assess variation in this parameter across plates by month.

**Table 1:** Summary of the counts for controls and samples included on all assay plates.

	HRP2	pAldolase			pLDH		PvLDH
Total number assay plates	504						
Plates repeated due to missing control data*	41						
**Plates with all control datapoints**							
Background	504	504				504	504
Negative	491	491				491	491
Positive 1:1	483	484				483	484
Positive 1:4	501	501				501	502
Positive 1:16	502	501				503	502
Positive 1:64	503	502				503	503
Plates with complete 4-point positive curve	477	476				476	479
Total number DBS tested	40,272						
Final number of observations for all samples by antigen	40,157		40,162	40,161			40,161

*Upon repeating assay plate, the previous control and sample data is replaced by repeat data.

### Ethics approval and consent to participate

Antigen detection in the previously collected samples was determined to be surveillance and not research by the office of the Associate Director of Science in the Center for Global Health at the Centers for Disease Control and Prevention, determined IRB review was not required by University of Maryland Baltimore, and provided a research exemption by National Health Research Ethics Committee (NHREC).

### Disclaimer

The findings and conclusions in this report are those of the authors and do not necessarily represent the official position of the Centers for Disease Control and Prevention and Nigeria Centre for Disease Control.

## Results

### Time of study and datapoints collected

Plasmodial antigen data were collected from November 2019 through March 2020 on DBS samples at the NRL. Every assay plate contained eight wells with controls: two (2) buffer blank, two (2) negative control blood, and a 4-point antigen positive dilution curve (1:1, 1:4, 1:16, 1:64) as described in “Materials and methods”. A total of 133 assay plates were completed in the first month, 79 in the second, 84 in the third, 114 in the fourth, and 94 in the fifth. From the 504 assay plates processed throughout the survey, 41 (8.1%) were immediately selected to be repeated due to loss of critical control information as described in “Materials and methods” (Table 1). After these 41 repeats, every plate provided background data (504/504) and 97.4% (491/504) provided negative control blood data. For the positive control dilution series, most plates successfully produced a complete 4-point dilution curve across the four antigens: 88.7% (447/504) for HRP2, 94.4% (476/504) for pAldolase, 94.4% (476/504) for pLDH, and 95.0% (479/504) for PvLDH. Similar robust data acquisition was given for the four antigen targets from the total number of Nigerian DBS samples collected (40,272): 99.7% (40,157/40,272) for HRP2, 99.7% (40,162/40,272) for pAldolase, 99.7% (40,161/40,272) for pLDH, 99.7% (40,161/40,272) for PvLDH. Data for all four antigen targets were viable for 99.7% of the DBS samples.

### Controls included on assay plates

**Figure 1:**
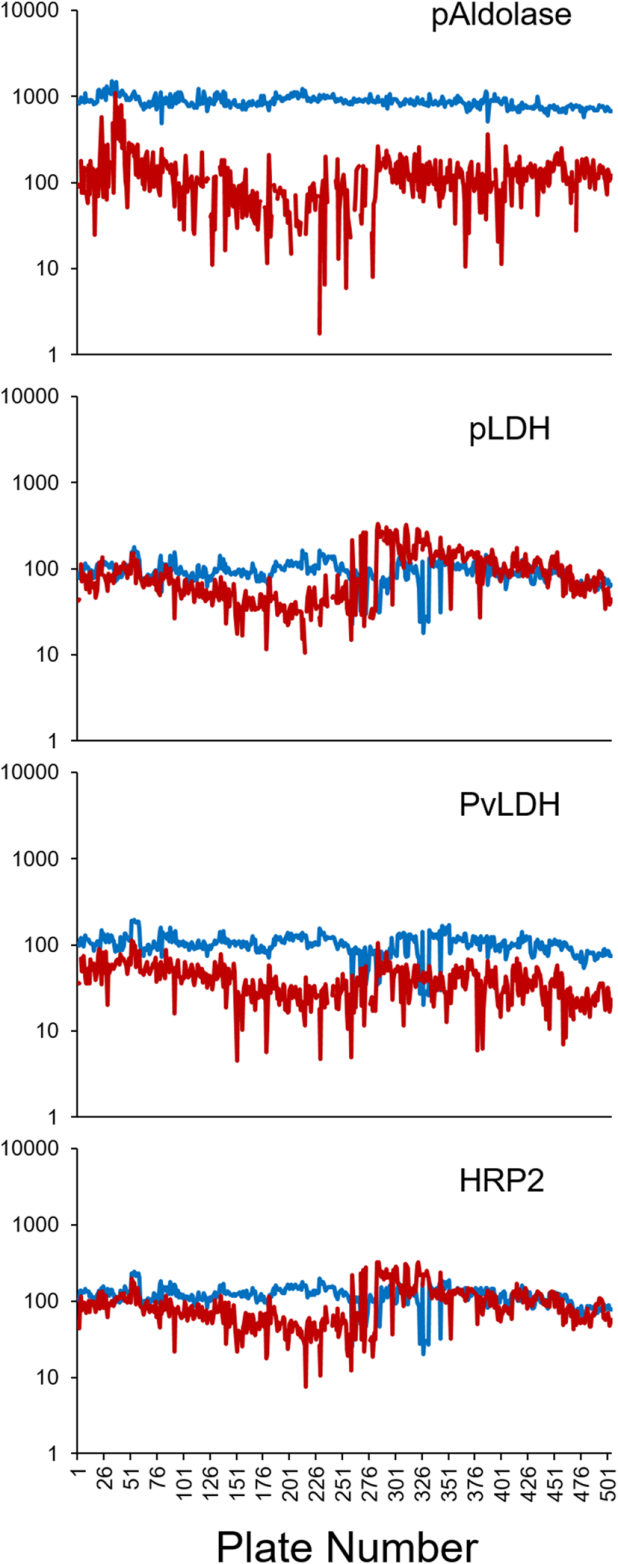
Assay signal values for buffer blank and antigen negative blood controls used through the study. Plate number shown as chronological sequence from first to last assay plate in the study. Assay signal displayed as the average median fluorescence intensity (MFI) for buffer blank duplicate (blue line) for each antigen target. Assay signal displayed as the average MFI minus buffer blank (MFI-bg) for the antigen negative blood duplicate (red line).

Assay signal averages for the buffer blank duplicate and negative blood duplicate on each plate were plotted over time (Fig. 1). These generally remained stable through the 5 months of laboratory data collection for all four antigens, though greater variation in signal response was seen in pAldolase negative control blood. Signals for the four different points of the positive control dilution series for each antigen across the dilution curve were also stable, though clearly showing a loss of assay signal over time (Supplementary Figure 1). The pAldolase signals for the positive control were highly variable and signal responses were negligible relative to the other antigens; therefore, pAldolase was not used as a determinant aberrant plate identification (Supplementary Figure 2).

### Criteria for assay plate repeat

**Figure 2:**
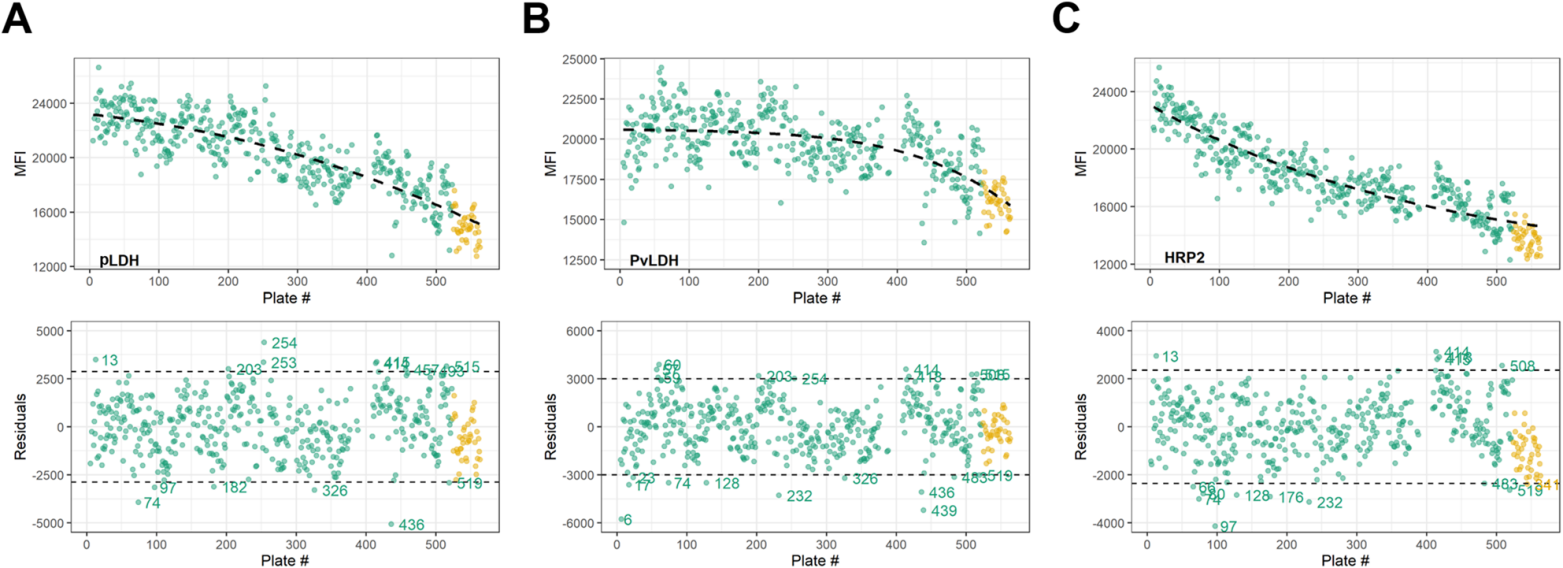
Assay signal values for positive control pool over time. Plots display regression functions for the first point of the positive control with plate number as the time variable throughout the course of data collection for the study. Regression plots displayed individually for pLDH (**A**), PvLDH (**B**), and HRP2 (**C**) antigens with residual plots shown directly below. For each plot, the 41 points shown in gold represent the assay plates repeated due to gross errors in positive or negative controls. For residual plots, assay plates with the first point positive signal outside 2 × SD are labelled.

To determine the plates requiring repeating based on the positive control signal, the first point of the positive control dilution series for pLDH, PvLDH, and HRP2 was fit to regression functions with assay signal plotted over time (Fig. 2). As shown by the residuals plots for different antigens in Fig. 2, if a plate’s control point was outside of 2 SD from the regression fitting for a particular antigen, that plate was flagged for potential concern. For a single plate, control points outside 2 SD for both pLDH and HRP2 were deemed outliers and qualified for repeating. As PvLDH is specific to *P. vivax*, a species sparsely found in Nigeria, this antigen’s positive control signal was not a criteria component identifying deviant plates. Fifteen (15) plates qualified as outliers for pLDH and 15 for HRP2 with only 6 assay plates (1.2% of all) showing outliers for both antigens (Table 2). Regression functions and residuals for the second (1:4) and third (1:16) positive control dilutions points are shown in Supplementary Figure 3.

**Table 2:** Plates with assay signals of first positive control above or below two standard deviations from regression curve.

Dispersion criteria	pLDH	HRP2	PvLDH*
+ 2 standard deviations above regression curve	**Plate 13** Plate 203 Plate 253 Plate 254 **Plate 414** **Plate 415** Plate 457 Plate 493 Plate 515	**Plate 13** **Plate 414** **Plate 415** Plate 418 Plate 508	Plate 57 Plate 59 Plate 60 Plate 203 Plate 254 **Plate 414** Plate 418 Plate 508 Plate 515
− 2 standard deviations below regression curve	**Plate 74** **Plate 97** Plate 182 Plate 326 Plate 436 **Plate 519**	Plate 66 **Plate 74** Plate 80 **Plate 97** Plate 128 Plate 176 Plate 232 Plate 341 Plate 483 **Plate 519**	Plate 6 Plate 17 Plate 23 **Plate 74** Plate 128 Plate 232 Plate 326 Plate 436 Plate 439 Plate 483 **Plate 519**

*Plates with PvLDH positive control outliers shown for reference, but not used in criteria for repeating an assay plate.

Plates for which both the pLDH and HRP2 signals were outliers are indicated in bold.

### Change in assay signal for positive control over time

**Figure 3:**
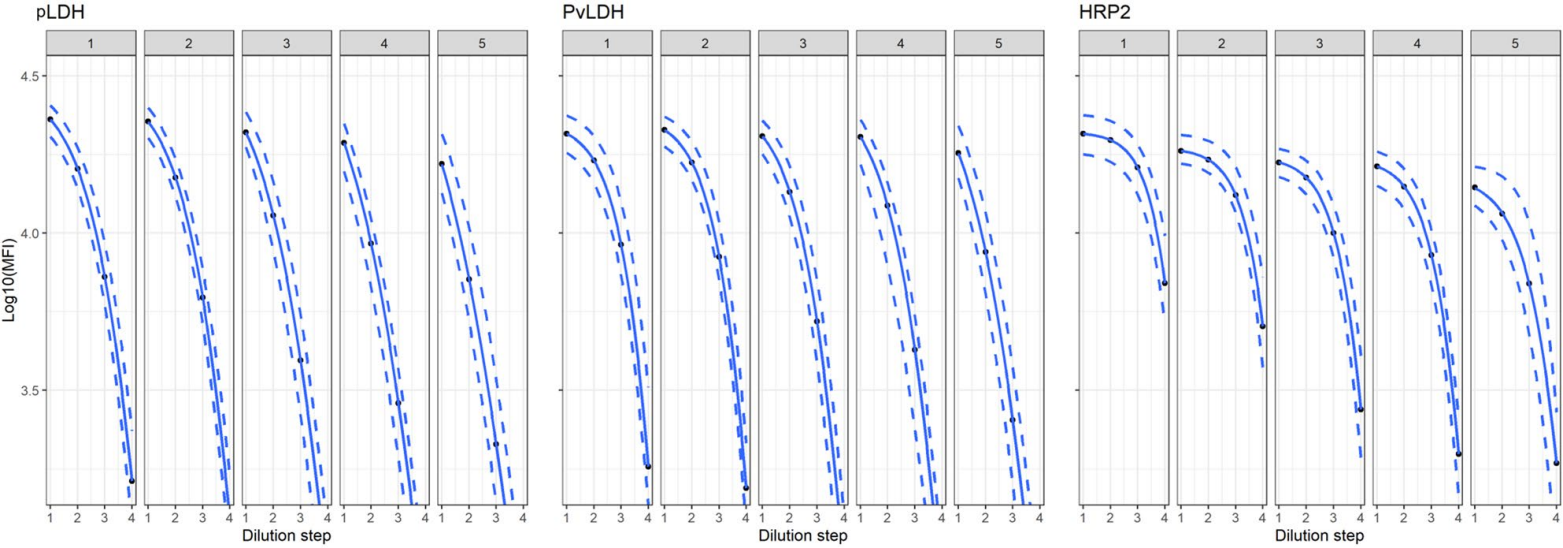
Regression fitting to the 4-point positive dilution series for pLDH, PvLDH, and HRP2 by month of data collection. Each individual plot displays the mean assay signal (solid line) with percentile dashed lines for 95 and 5% extending above and below each solid line, respectively. For each antigen, five plots are shown for assay data collected within each of the 5 months.

The 4-point positive control dilution curves by month are shown in Fig. 3 for all antigens except pAldolase. As shown by Fig. 3 and Supplementary Figure 1, positive control signals for all antigens decreased over time with HRP2 showing the greatest decrease over the 5 months of testing. Inspection of the *xmid* coefficient and coefficient of variation (%CV) was used to assess variation within the positive dilution curves (Supplementary Figure 4). The %CV for the antigens remained below 5% throughout the survey except for HRP2 during the fifth month, which exhibited relatively higher %CV at 11%. Across all antigens, a higher %CV was seen in month 5 relative to earlier months.

### Antigen standard curves over time

**Figure 4:**
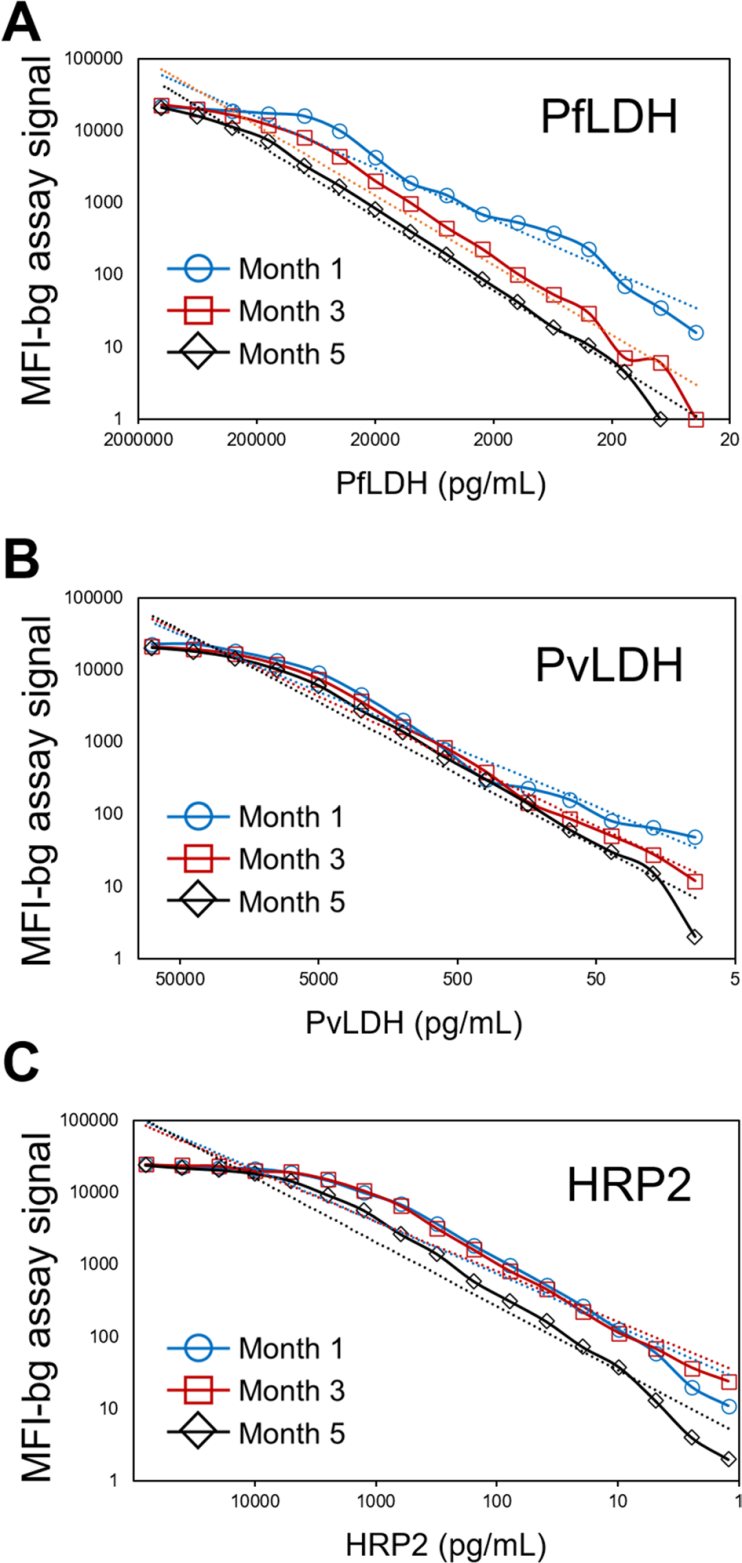
Recombinant antigen standard curves throughout duration of data collection for study. Standard curves were run at months 1 (blue line with circles), 3 (red line with squares), and 5 (black line with diamonds) for PfLDH (**A**), PvLDH (**B**), and HRP2 (**C**). Hashed lines for each dilution series indicate regression fitting to power equation (y = Ax^b^; regression estimates shown in Supplementary Table 1).

At months 1, 3, and 5, complete standard curves were prepared for PfLDH, PvLDH and HRP2 and run separately from the assay plates containing blood samples. Comparison of these twofold serial dilution curves over the 3 months is shown in Fig. 4 and regression estimates shown in Supplementary Table 1. Slight decreases in assay signal over time were noted for all three antigens but were most pronounced for PfLDH.

## Discussion

We outline here a quality assurance scheme for inter- and intra- plate variability for multiplex malarial antigen detection which would be appropriate for large sample sets. RDT-based antigen detection tests have been utilized by the malaria community as a key component for monitoring population-level estimates of malaria^3,5^. While field-deployable RDTs were designed to reliably detect higher antigen concentrations and are able to provide a fast diagnostic result, antigen detection through sensitive quantitative immunoassays is valuable for determination of antigen concentrations in blood samples and further epidemiological and research investigations^1^. Antigen data gained through these sensitive laboratory assays is cost-effective compared to PCR assays, and has been used to evaluate RDT field performance^18^, as well as estimate *Plasmodium* infection prevalence and presence of *pfhrp2* deletions^8,12^. Surveys sampling larger percentages of a study population will inherently provide more accurate and precise estimates of malaria in a population, so to capture these accurate estimates for large-scale surveys, laboratories must possess the ability to not only collect and process large volumes of samples in a timely manner, but also efficiently implement quality assurance activities to ensure data validity and reliability.

In this study, bead-based multiplex antigen detection was performed on a large number of samples stored in a biorepository in Nigeria. Antigen data collection ran from November 2019 through March 2020, and over the 5 months of sample collection 504 plates containing 40,272 participant samples were tested. The rate of running assay plates was on average 101 plates/month (range 79–133/month) which provided data on approximately 8000 samples/month. The multiplex protocol was implemented for the detection of pLDH, PvLDH, HRP2, and pAldolase antigens enabling high throughput data collection for this study. The large sample set made it imperative to include controls to verify plate consistency, and the quality assurance schema utilized here assessed inter-plate variability and assay validity based on positive and negative controls included on every plate. Though the current study aimed to define a multi-antigen algorithm for assay plate pass/failure, additional strategies based on control values on each assay plate could also be used in future studies to normalize or adjust sample data post hoc. As very few positive controls were included on the assay plates for this study, this type of data adjustment was not attempted since an aberrant signal for any of the antigen controls could substantially affect all sample data on that assay plate.

Assay consistency was assessed using negative and blank assays signals over time for each antigen. Signals for these antigen negative samples were observed to be stable and variability was limited between plates for pLDH, PvLDH, and HRP2 antigens; however, greater variation in negative blood signals were observed for pAldolase. It is unclear why the pAldolase negative signal provided such a high degree of variation, though this has been noted by previous work our group has done and may be due to the polyclonal nature of this antibody clone^8^. Given this variability in assay signal for negative controls, as well as inconsistencies in pAldolase positive control values over time (Supplementary Figure 2), further analyses utilizing pAldolase data will be restricted to binary classification (antigen +/−) rather than attempting to quantify pAldolase concentrations.

Regression functions and residual plots of the positive controls were used to assess inter-plate variability. The regression functions showed a decrease in positive assay signal over time exhibiting the most profound decrease in HRP2 (Fig. 2). At the beginning of the study, a large batch of the positive 4-point dilution series was prepared with the intent of using throughout the entire study, and the loss in signal could be attributed towards the antigen in the positive pool degrading over time. When evaluating the standard curves of positive control blood produced at months 1, 3, and 5 of the study, decreases in signal were observed over time, but at a rate lesser than what was observed in the positive controls collected in Nigeria. This observation supports the explanation that the reduction of signal in the positive controls may be ascribed to antigen degradation, but also supports the concept that assay reagents may lose reactivity over time as well. As the standard curves allow extrapolation between assay signals and antigen concentration for the plates processed over the course of the survey^8,17^, DBS assay data will be able to be matched to the time period the standard curve was run to allow the most accurate extrapolation between signal and concentration. Future studies should also test if aliquoting and freezing preparations of recombinant antigen pools to allow for intermittent preparation of positive controls (as a single point or a curve) would serve to better retain the absolute assay signal over the course of data collection. Additionally, other types of positive controls such as preparations from parasite cultures or purified native antigens may retain better stability over time.

Residual plots were used to identify antigen positive control signals that showed greater deviation flagging them as potential outliers. For this study, plates with positive controls that fell outside the mean ± 2 SD of signals at the first point of the dilution series for pLDH and HRP2 qualified to be repeated. The rationale to exclude PvLDH from the criteria was that this particular species has a very low prevalence in Nigeria. After applying the outlier criteria to the 504 plates collected, 6 (1.2%) plates were identified for requiring repeat analysis. This is in addition to 41 (8.1%) of assay plates that needed to be repeated due to absence of critical information about blank wells or other controls on the plate. As assay data can be observed in real-time on the MAGPIX system, checking the progression of each assay plate as it is being run on the system can allow users to automatically suspend a plate run and immediately repeat if inconsistencies are noted in control wells. This operating procedure of immediate assessment and repeating was not initially set up for this first study of 504 plates but will be utilized for future sample sets to allow greater efficiency in the laboratory. The proposed methodology presented here allows for systematic assessment of variation among assay plates to ensure consistency in data collection over the course of a multiplex antigen detection study and can be modified as needed for other studies and laboratories for their unique circumstances.

## Supplementary Information

Supplementary Information.

## Data Availability

NAIIS technical report: Federal Ministry of Health, Nigeria. Nigeria HIV/AIDS Indicator and Impact Survey (NAIIS) 2018: Technical Report. Abuja, Nigeria. October 2019. All data are available from the authors upon request.
