# Supplementary material for: Considerations for quality assurance of multiplex malaria antigen detection assays with large sample sets
Source: Sci Rep. 2021 Jun 24;11:13248. doi: 10.1038/s41598-021-92723-w (PMC8225651; doi:10.1038/s41598-021-92723-w)
Supplement: Supplementary file 1 — Supplementary Information. [file 41598_2021_92723_MOESM1_ESM.pdf]

Supplementary Material for:

Considerations for Quality Assurance of Multiplex Malaria Antigen Detection Assays with Large Sample Sets

Rachel Alvarado[1], Lotus L. van den Hoogen[2], Nnaemeka C. Iriemenam[3], Oluwaseun O. Akinmulero[4], Andrew N. Thomas[4], Israel Tamunonengiyeofori[5], Evbuomwan Erasogie[5], Achugbu C. Chimaoge[5], Ayuba B. Dawurung[4], Mudiaga K. Esiekpe[4], Mary U. Okoli[5], Nwando Mba[5], Abiodun Ogunniyi[5], Alash'le Abimiku[4], Mark Maire[1], Orji O. Bassey[3], McPaul Okoye[3], Mahesh Swaminathan[3], Stacie M. Greby[3], Nnaemeka Ndodo[5], Chikwe Ihekweazu[5], Ado Abubakar[4], Laura Steinhardt[1], Eric Rogier[1#]

[1] Malaria Branch, Division of Parasitic Diseases and Malaria, Centers for Disease Control and Prevention, Atlanta, GA, USA

[2] Center for Applied Malaria Research and Evaluation, Tropical Medicine Department, Tulane University School of Public Health & Tropical Medicine, New Orleans, LA, USA

[3] Division of Global HIV & TB, Centers for Disease Control and Prevention, Abuja, Nigeria

[4] Institute of Human Virology (IHVN), Central Business District, Abuja, Nigeria

[5] Nigeria Centre for Disease Control (NCDC), Abuja, Nigeria

[#]Please address all correspondence to Eric Rogier, Malaria Branch, Centers for Disease Control and Prevention, Atlanta, Georgia, USA; erogier@cdc.gov

Supplementary Table 1. Regression estimates for standard curves created with recombinant antigens by month.

		Regression: $y=Ax^b$		
**Antigen**	**Date**	**Estimate for A**	**Estimate for b**	**R^2**
PfLDH	Month 1	2.49	0.72	0.96
	Month 3	0.09	0.97	0.97
	Month 5	0.03	1.02	0.99
PvLDH	Month 1	5.66	0.8	0.97
	Month 3	2.08	0.9	0.98
	Month 5	0.72	1.0	0.97
HRP2	Month 1	26.2	0.73	0.93
	Month 3	32.7	0.70	0.94
	Month 5	4.46	0.89	0.95

Supplementary Figure 1

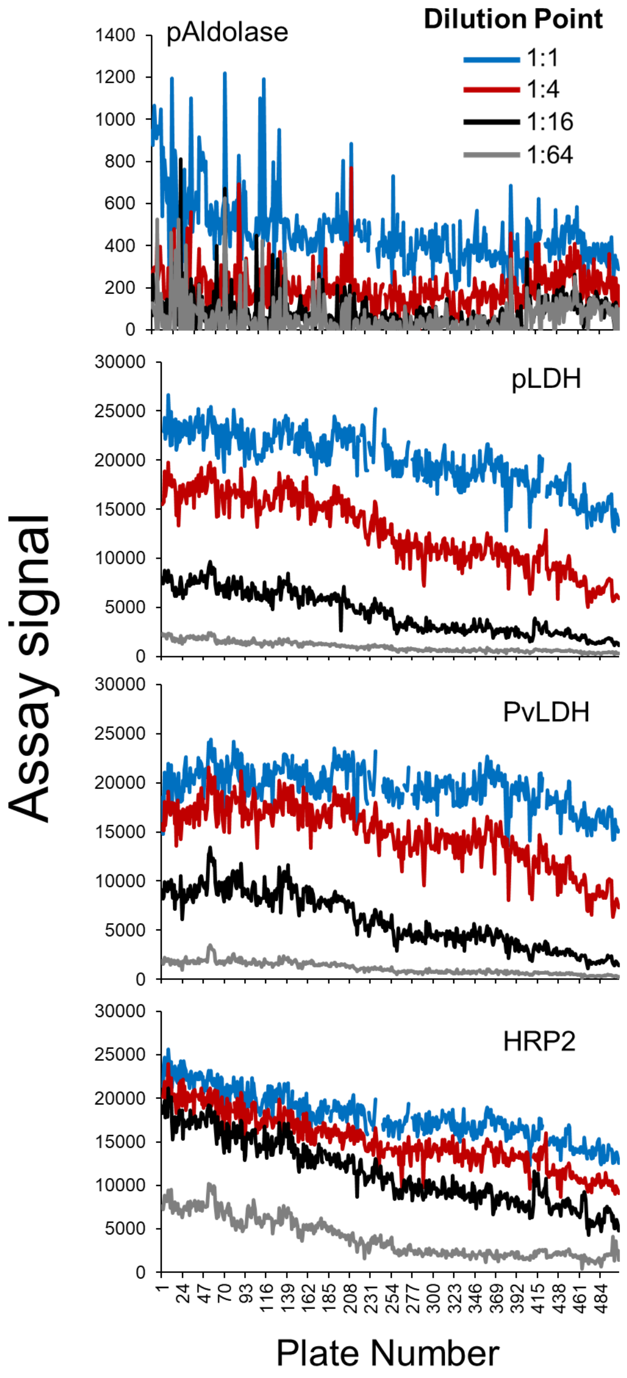

Supplementary Figure 1. Assay signal for the four points of the positive control dilution series per assay plate. For each antigen's MFI-bg signal, lines connect dilution points of 1:1 (blue), 1:4 (red), 1:16 (black), and 1:64 (grey).

Supplementary Figure 2

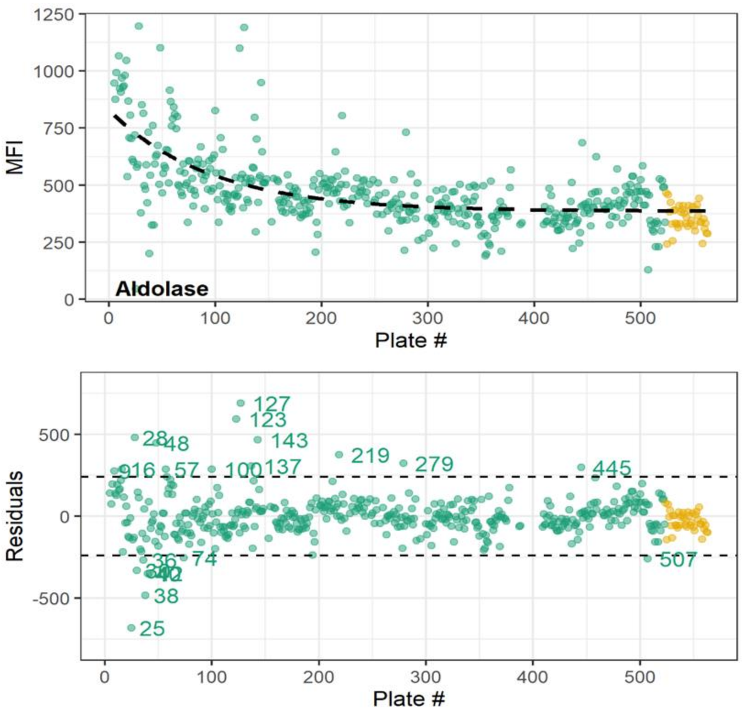

Supplementary Figure 2. Assay signal values for positive control pool over time for pAldolase. Plots display regression functions for the first point of the positive control with plate number as the time variable throughout the course data collection for the study. Regression plots displayed for pAldolase with residual plot shown directly below. For residual plot, assay plates with the first point positive signal outside 2*SD are labelled.

Supplementary Figure 3

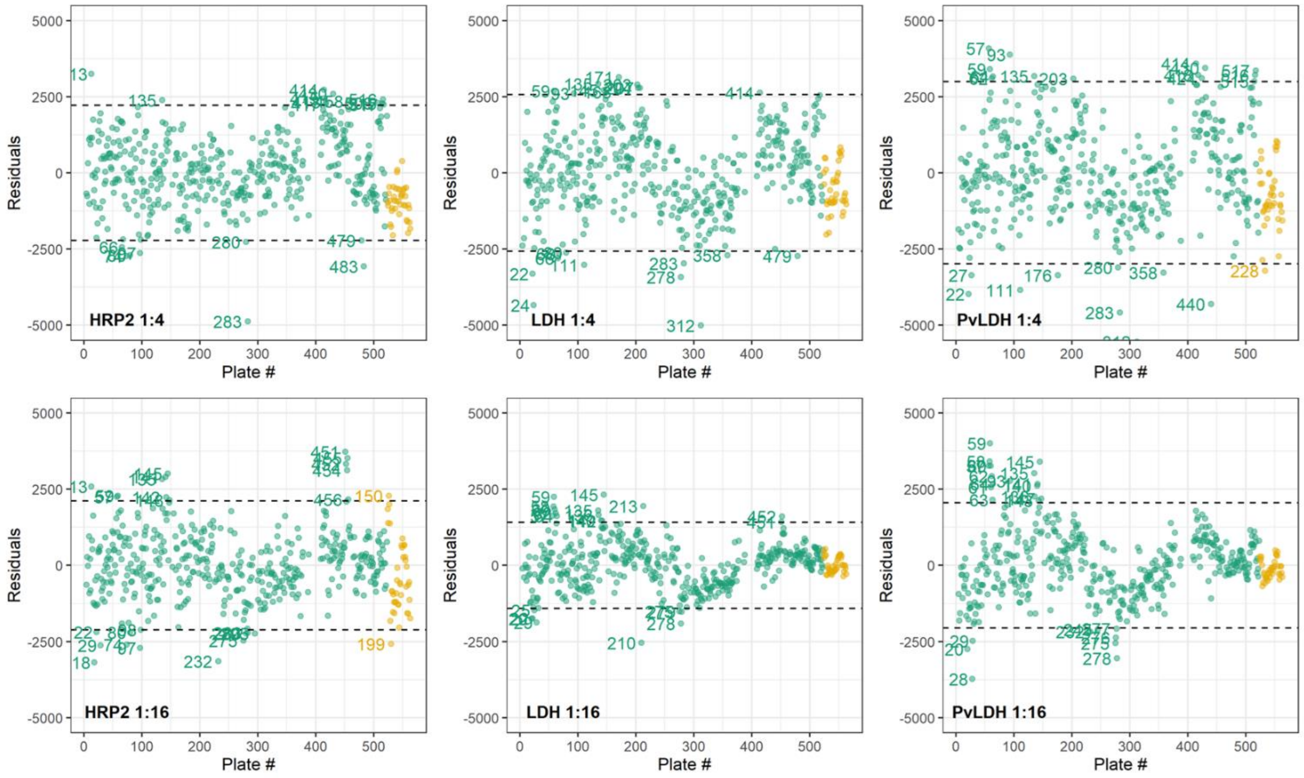

Supplementary Figure 3. Assay signal values for positive control pool over time for the second and third points. Plots display residuals for the regression functions for the second (1:4) and third (1:16) points of the positive control with plate number as the time variable throughout the course data collection for the study. Assay plates with the first point positive signal outside 2*SD are labelled

Supplementary Figure 4

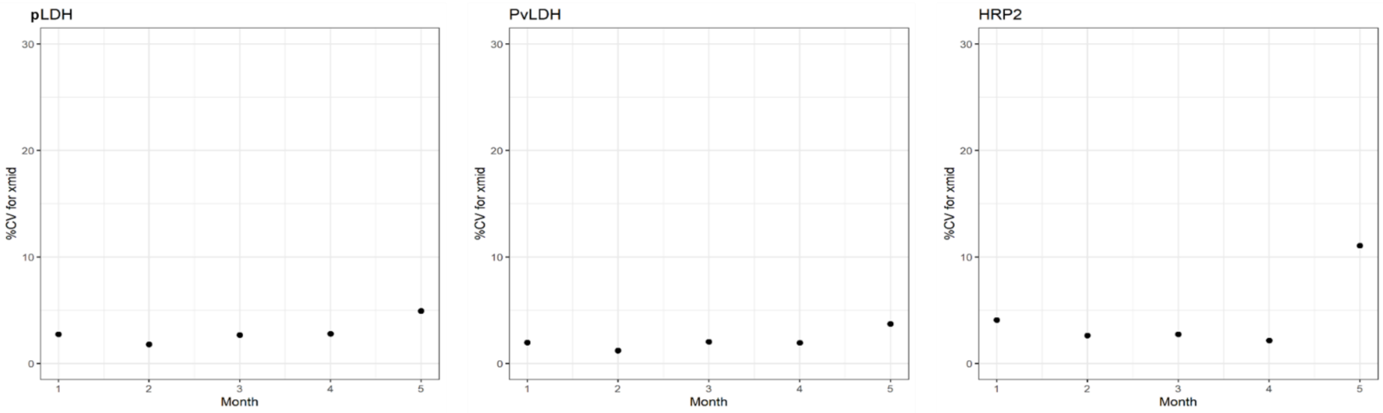

Supplementary Figure 4. The coefficient of variation for the *xmid* coefficient from 3-parameter logistic regression for pLDH, PvLDH, and HRP2 by month of data collection.
